# Neurophysiology and Psychopathology Underlying PTSD and Recent Insights into the PTSD Therapies—A Comprehensive Review

**DOI:** 10.3390/jcm9092951

**Published:** 2020-09-12

**Authors:** Gjumrakch Aliev, Narasimha M. Beeraka, Vladimir N. Nikolenko, Andrey A. Svistunov, Tatyana Rozhnova, Svetlana Kostyuk, Igor Cherkesov, Liliya V. Gavryushova, Andrey A. Chekhonatsky, Liudmila M. Mikhaleva, Siva G. Somasundaram, Marco F. Avila-Rodriguez, Cecil E. Kirkland

**Affiliations:** 1 GALLY International Research Institute, 7733 Louis Pasteur Drive, #330, San Antonio, TX 78229, USA; 2 I.M. Sechenov First Moscow State Medical University of the Ministry of Health of the Russian Federation (Sechenov University), 8/2 Trubetskaya Str., 119991 Moscow, Russia; vn.nikolenko@yandex.ru (V.N.N.); svistunov@mma.ru (A.A.S.); stm-i@yandex.ru (T.R.); cherkesovi@gmail.com (I.C.); 3 Research Institute of Human Morphology, 3 Tsyurupy Street, 117418 Moscow, Russia; mikhalevalm@yandex.ru; 4 Institute of Physiologically Active Compounds of Russian Academy of Sciences, Severny pr. 1, Chernogolovka, 142432 Moscow Region, Russia; 5 Department of Biochemistry, Center of Excellence in Regenerative Medicine and Molecular Biology (CEMR), JSS Academy of higher education and Research (JSS AHER), Mysuru 570015, Karnataka, India; bnmurthy24@gmail.com; 6 Department of Normal and Topographic Anatomy, M.V. Lomonosov Moscow State University, Leninskie Gory, 1, 119991 Moscow, Russia; 7 Research Centre for Medical Genetics (RCMG), Moscow 119991, Russia; svet-vk@yandex.ru; 8 Saratov State Medical University named after V. I. Razumovsky, Bolshaya Kazachya str., 112, 410012 Saratov, Russia; gavryushova.liliya@yandex.ru (L.V.G.); fax-1@yandex.ru (A.A.C.); 9 Department of Biological Sciences, Salem University, Salem, WV 26426, USA; siva15ram58@gmail.com (S.G.S.); EKirkland@salemu.edu (C.E.K.); 10 Department of Clinic Sciences, Faculty of Health Sciences, University of Tolima, Barrio Santa Helena, Ibagué 730006, Colombia; markos.avila@gmail.com

**Keywords:** neuroinflammation, neuroendocrine, neurogenesis, PTSD-treatment, neurotransmitters, SSRIs

## Abstract

Post-traumatic stress disorder (PTSD) is a well-known psychiatric disorder that affects millions of people worldwide. Pharmacodynamic and cognitive-behavioral therapies (CBT) have been used to treat patients with PTSD. However, it remains unclear whether there are concurrent changes in psychopathological and neurophysiological factors associated with PTSD patients. Past reports described those PTSD patients with efficient fatty acid metabolism, neurogenesis, mitochondrial energy balance could improve ability to cope against the conditioned fear responses and traumatic memories. Furthermore, cognitive, behavioral, cellular, and molecular evidence can be combined to create personalized therapies for PTSD sufferers either with or without comorbidities such as depression or memory impairment. Unfortunately, there is still evidence lacking to establish a full understanding of the underlying neurophysiological and psychopathological aspects associated with PTSD. This review has extensively discussed the single nucleotide polymorphism (SNPs) of genetic factors to cause PTSD, the implications of inflammation, neurotransmitter genomics, metabolic alterations, neuroendocrine disturbance (hypothalamus-pituitary-adrenal (HPA) axis), mitochondrial dynamics, neurogenesis, and premature aging related to PTSD-induced psychopathology and neurophysiology. In addition, the review delineated the importance of CBT and several pharmacodynamic therapies to mitigate symptomatology of PTSD.

## 1. Introduction

Post-traumatic stress disorder (PTSD) comprises a set of alterations in cognition and mood [1]. PTSD is associated with a devastating constellation of symptoms resulting from persistent and prolonged exposure to traumatic events that directly or indirectly evoke stress [2]. PTSD has been reclassified in the Diagnostic and Statistical Manual 5th edition (DSM-5). It is no longer categorized as an anxiety disorder but categorized as “Trauma- and Stress-or-Related Disorder”. This reclassification was engendered by the adverse effects in common with other stress-related disorders as well as new diagnostic criteria [3,4]. According to DSM-5, the diagnostic criteria to classify PTSD are exposure to actual or threatened death, serious injury, or sexual violation. According to the American Psychiatric Association, the exposure must result from one or more of the following scenarios in which the individual:

- Directly experiences the traumatic event;
- Witnesses the traumatic event in person;
- Learns that the traumatic event occurred to a close family member or friend (with the actual or threatened death being either violent or accidental); or
- Exposed extensively to aversive traumatic events but specifically not through television, pictures or media.

The above list is not exhaustive. There are other examples of traumatic events viz., violent crimes, accidents, emotional-social abuse, physical assault, military combat, civil unrest, natural disasters, child abuse that can lead to PTSD in some individuals. Thus, PTSD is referred as a psychiatric disorder that may be characterized by clinically significant social impairment, incapacity to work, or diminishing mental ability to perform other daily functions [4]. Events that are experienced as traumatizing by the individual cannot be disputed. There are no thresholds or benchmarks to judge whether an event is sufficiently traumatic to trigger PTSD. Currently, about 24 million people are diagnosed with PTSD in the United States (or about 8% of the population). Furthermore, the public costs to treat PTSD has been increased almost US$43 billion per year [3].

PTSD is characterized by a spectrum of psycho-emotional and neurophysiological effects, such as re-experiencing the trauma in the form of vivid intrusive memories, flashbacks, or nightmares [3]. These experiences often are followed by overwhelming fear and strong physical sensations. PTSD sufferers often attempt to suppress memories or avoid activities reminiscent of the traumatic event(s) withdrawing from society. PTSD sufferers also report a heightened sense of fear of current threats, for example, hyper-vigilance and excessive reactions to unexpected noises. These symptoms noticeably impair personal, family, social, educational, occupational, and other important areas of functioning and quality of life [3,4].

Neurophysiological abnormalities are associated with imbalance in the functional aspects of hypothalamus-pituitary-adrenal (HPA) axis [5], altered immune, neurotransmitter, and neurotropic functions [1], increased thyroid activity [6], high nervous system sensitization, accelerated aging processes due to increased DNA damage, and telomere shortening [1]. In addition, PTSD is associated with osteoporosis, migraine, sleep disorders, respiratory disorders, cardiovascular disease, autoimmune diseases, chronic inflammation, metabolic syndrome, and early death from unknown causes [7,8].

The psychopathology and pathophysiology of PTSD are associated with deep-seated feelings or memories of the traumatic event that remain vivid. The memories do not simply fade over time but may linger or even intensify for years [3]. These effects may be related to the progressive damage inside CNS that facilitates the incidence of chronic PTSD. A smaller hippocampus in PTSD patients may be a cause for the development of stronger perceptions of fear and acquisition of avoidance feelings related to auditory cues paired with shock [9,10]. Furthermore, hippocampal volume is correlated with fear-mediated performance and may predispose the individual to have diminished neuroendocrine function via HPA axis. This is evident in many individuals with PTSD who endured childhood trauma due to higher cortisol levels [11]. Thus, a small hippocampal volume is associated with psychopathological changes and may predispose patients to acquire persistent conditioned psycho-emotional responses to hormone-induced stressful cues [12,13].

Several pathophysiological changes have been reported in PTSD patients and these changes are reported to be overlapped with the clinical manifestations observed in traumatic brain injury (TBI) patients [6]. In addition, the pathophysiological changes in amygdala, hippocampus, and associated structures in the brain are associated with PTSD [14,15,16,17]. The underlying networks from these brain regions with *parahippocampal gyri* and visual processing stream are reported to be involved in processing traumatic information and recalling traumatic memories [18,19]. Involuntary memory intrusions possibly are mediated by processing of traumatic visual memories [20,21,22].

### Diagnosis and PTSD

Brain scanning using single-photon emission computerized tomography (SPECT) may contribute to diagnosis of PTSD with high accuracy compared to other MRI and CT scans, which often obtain normal results from PTSD patients. SPECT imaging has been shown to differentiate PTSD from traumatic brain injury (TBI) of varying degrees of severity in large patient cohorts. SPECT scans could reveal relative increase in perfusion across limbic regions, basal ganglia, thalamus, and temporal lobes of PTSD patients compared to subjects with TBI [23]. Clinicians should seek the most accurate diagnostic approaches to choose suitable PTSD-targeted therapeutic options [22]. Moreover, brain tissue recovery is enhanced for PTSD patients who interact socially to gain new learning skills, engage in regular exercise, avoid potential threats and negative thoughts, and enthusiastically develop skills to execute challenging tasks [1,3]. Such healthy lifestyles are complemented by nutrition and weight management, good sleep patterns, and regular medical supervision.

Brain imaging studies have reported that individuals with PTSD have a hyperactive prefrontal cortex (PFC) and an underactive amygdala when compared to controls [24]. These findings suggest that inhibitory neuronal signaling loops mediating fear management are disrupted in PTSD patients experiencing traumatic memories [25]. Neuroimaging studies of PTSD patients revealed hyperactivity of limbic and medial brain pathways in generating an exaggerated response to even low-level social threat signals that, in turn, may be a major factor in emotional recognition and mentalization [26].

Currently, there are a very limited number of drugs approved for PTSD therapy. However, patients with PTSD generally are prescribed a number of drugs. This is related directly to their heterogeneous symptoms viz., chronic pain, anxiety, and fibromyalgia [27,28,29]. Abnormalities in the endogenous opiate system for intrinsic pain management are evident in PTSD patients [30,31]. The PTSD symptoms were prominently higher among the patients who received analgesics when measured using the *Posttraumatic Symptom Scale*. Astudy by Metzger, Linda J. et al., 1999 delineated the chronic PTSD-induced psychopathological effects in the victims of childhood sex abuse (CSA); they have shown an enhanced eye blink and autonomic reactivity to the startling tones than the CSA victims without PTSD, suggesting a clinical improvement with respect to the persistent biological traits underlying chronic PTSD symptomatology [32]. However, it is imperative to develop novel diagnostic approaches, therapeutic interventions and cognitive-behavioral therapies (CBT) to achieve clinical remissions in the PTSD patients.

In this review, we describe the changes in the body associated with PTSD, such as inflammation, dysregulation of the immune response, changes at the level of neurotransmitters, metabolic changes, HPA axis imbalances, and others. Further, we elucidate the causes of PTSD that have been reported in the literature and formulate a proposed coordinated sequence of events that may provide a basis for developing novel drug therapies to address adverse neurophysiological and psychopathological changes.

## 2. Genetics and PTSD

It has been reported that there is a significant genomic relationship between PTSD and single nucleotide polymorphism (SNP) in the protein-coding gene ANKRD55. Genome-wide association studies (GWAS) of PTSD patients from African-American origin have exhibited SNP on the intron sequences of ANKRD55 gene. This SNP appears to have a significant clinical relevance to the genomic wide association of PTSD with several autoimmune and inflammatory disorders in African-American patient samples but not White patient samples [33,34,35]. However, the function of this gene is yet to be fully explained in the pathophysiology of PTSD. This genetic link has been correlated to a range of autoimmune and inflammatory disorders including multiple sclerosis, type 2 diabetes, celiac disease, and rheumatoid arthritis [34,36].

Veterans of the Vietnam War (1955–1975) experienced PTSD were demonstrated by Pitman et al. [37] and Gilbertson and colleagues [13]. They report that twins who were exposed to war trauma and developed PTSD have significantly smaller hippocampi. These reports concluded that the smaller hippocampi may be due to genetic, neurodevelopmental, or multifactorial vulnerabilities that predisposed these individuals to develop PTSD [13,37]. Reduced size of the hippocampus has not been reported as being due to PTSD. Twin studies have shown a moderate hereditary component of PTSD (in the range of 30–50%) and a high risk of traumatic effects (in the range of 30–60%), along with the genetic and environmental influence for PTSD incidence [37]. The underlying mechanism of these genetic effects in conjunction with traumatic exposure leading to PTSD remains unclear [38].

GWAS studies in military veterans affected with PTSD have concluded that there is a significant whole-genome association between PTSD and SNP *rs8042149* gene that code for retinoid-related orphan receptor alpha (RORA) [39]. SNP studies of these individuals were performed to ascertain PTSD symptoms after hurricane exposure. This study revealed a specific relation to the severity of PTSD symptoms [39]. These findings are encouraging for neurobiologists to delineate the genetics of factors underlying the traumatic stress because the psychopathology is interlinked with 606 SNPs spanning the RORA gene in PTSD patients [39,40,41].

Estrogenic status may increase the risk of PTSD in some women partly due to the methylation of Histone deacetylase 4 (*HDAC4)*. It is a protein responsible for several critical functions such as transcriptional regulation, cell cycle progression, and developmental events. The observation of higher levels of *HDAC4* methylation in PTSD cases suggests that there is a role of this protein in the onset or progression of PTSD that still to be unraveled. In traumatized individuals, lower *HDAC4* expression is reported to worsen fear sensitivity and intensity [42]. Another study reported a significant variation in the *ADCYAP1R1* gene encoding for ‘pituitary adenylate cyclase-activating polypeptide type I receptor’ associated with PTSD in women. In particular, SNP *rs2267735* gene in the putative estrogen response element (ERE) was associated with the acquisition of PTSD through the gene-environment interactions. Although genomic analyses have not confirmed the main effect of this gene in PTSD, the gene-environment interactions for the underlying SNPs have been observed in women patients with childhood adversity and prolonged trauma [43].

## 3. Inflammation and PTSD

**Figure 1:**
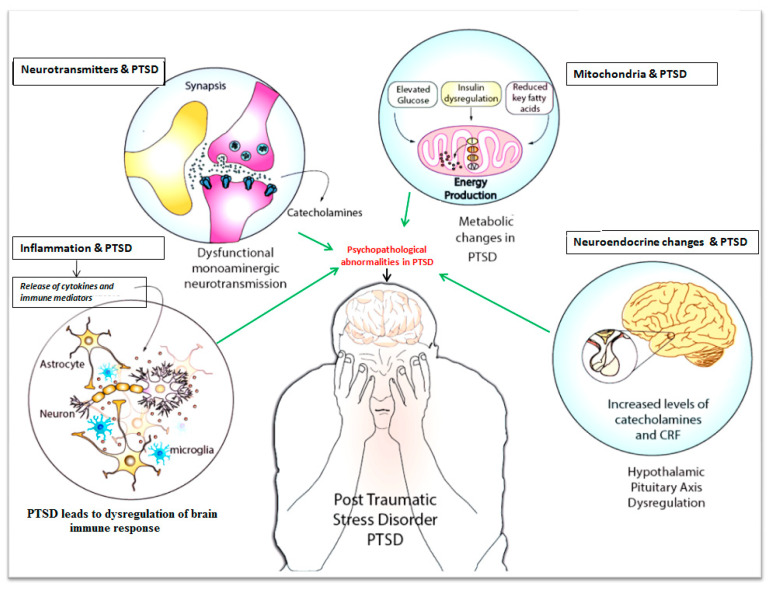
Systemic changes correlated to psychopathological changes during post-traumatic stress disorder (PTSD): PTSD may be associated with multifactorial disorders in the human body. A dysregulation of the immune system fosters neurophysiological and psychopathological changes due to elevated pro-inflammatory mediators such as IL-6 and IL-17. Impaired balance in the catecholamine neurotransmitter levels (e.g., monoaminergic neurotransmitters) across the synaptic junction confers psychopathological changes in PTSD. Metabolic alterations and mitochondrial dysfunction in neurons foster the psychopathological andneurophysiological changes during PTSD. Balanced release of secretions through hypothalamus-pituitary-adrenal (HPA)-axis signaling is crucial for ameliorating the PTSD effects since HPA-axis dysregulation causes inflammation, insulin resistance, oxidative stress, and energy deficiency due to the increased levels of catecholamines and corticothropin release factor.

PTSD is associated with dysregulation of the immune response, which is reflected by an increase in pro-inflammatory cytokines, namely interleukins viz., IL-6 and IL-17, and a decrease in IL-4 [31,44,45]. Further, there are findings that support links between IL-6 and HPA axis dysregulation to foster PTSD symptoms [46,47] (Figure 1). Glucocorticoid secretions through HPA axis typically inhibit the lymphocyte proliferation and reduce the secretion of pro-inflammatory cytokines viz., IL-6, IL-12, interferon γ (IFN-γ), and tumor necrosis factor α (TNF-α) during stress conditions [47]. In addition, the gut microbiome plays a vital role in programming endocrine HPA axis, a key stress response regulator [48,49]. Dysregulation in the HPA axis is associated with pathophysiology of PTSD [50]. A recent study found that a heat-killed preparation of an immune-regulatory bacterium significantly enhanced the Treg anti-inflammatory cytokines including IL-10 and tumor growth factor-β (TGF-β) during stress cues incurred in PTSD of mice models [51]. Imbalance between inflammatory cascades or immuneregulation by microbial input could increase the risk of acquiring PTSD-like syndrome [52].

A plethora of scientific reports has described the enhanced activity of IL-6 during stress, which progressively induces an extensive rise in the neuronal activity of the amygdala in response to stress [47]. Additionally, the severity of PTSD symptoms has been correlated with IL-6 levels [53]. Elevated IL-6 levels can cause mood disorders and enhance the activity of subgenual anterior cingulate cortex (ACC). IL-6-induced mood changes with underlying neuroinflammation have been linked to the reduced connectivity of subgenual ACC with other areas of the brain [47]. IL-6, IL-1β, and TNF-α have been reported to influence the CNS to damage cognitive levels by affecting neurogenesis, learning and memory, and synaptic plasticity [45,54]. Passos et al. (2015) found that the IL-1 and IL-6 levels remain elevated in PTSD patients even when comorbid depression patients were excluded from the research [45,55]. However, variations in the genes coding for the inflammatory factors may be part of PTSD predisposition. Future research should focus on the development of therapeutic modalities to mitigate the inflammation-mediated mood changes during PTSD.

The Grady Trauma Project at Emory University in Atlanta, Georgia, USA studied high PTSD rates among predominantly African-American urban residents. This study outlined a significant link between PTSD and variations in the pro-inflammatory C-reactive protein (CRP) gene [46,56]. The levels of CRP are correlated positively with PTSD symptoms. Vasiliki M et al. (2014) reported SNPs in CRP gene or the higher CRP levels associated with fear, avoidance, and other PTSD symptoms mainly among the traumatized patients [46]. This report concluded that the substantial rise in pro-inflammatory cascade and CRP levels may be involved in the fear-related PTSD psychopathology and heightened arousal symptoms [46]. Therapeutic modalities to mitigate the pro-inflammatory cascades across neurons and neuroglia may benefit PTSD patients.

Typically, stress can induce new blood cell formation [57]. Several blood cell types are normally elevated in PTSD. For example, RBC, WBC, and platelet counts were significantly higher in combat-related PTSD patients; and platelet count is significantly related to delineate the severity of PTSD. Inflammation could induce dysregulation in the hematopoietic stem cell lineages and consequently fosters production of various other blood cells in combat-related PTSD patients [58,59].

## 4. Neurotransmitter Genomics and PTSD

Genes pertaining to the monoaminergic neurotransmission associated with PTSD have been studied extensively. The most widely demonstrated polymorphism is a ‘tandem variable repeat (VNTR)’ in the promoter region of the serotonin transporter gene (SLC6A4), which encodes the target protein for ‘the activity serotonin re-uptake inhibitors’ implicated in PTSD treatment [60]. The functional polymorphism in *5HTTLPR* gene is related to the incidence of stress-related psychiatric disorders; this polymorphism spanning the *5HTTLPR* is common in European populations with a frequency of approximately 45% for the so-called short allele [60]. Further, this kind of genetic polymorphism could facilitate diminished transcription of SLC6A4 [60]. For example, people who have experienced traumatic events are at a higher risk of acquiring PTSD and they exhibit lower methylation levels at the SLC6A4 locus. Such results contribute to the conjecture that the methylation status of SLC6A4 interacts with brain’s processing of the traumatic experiences consequently enhance the risk of developing PTSD [61,62].

A study by Nadia Solovieff et al., 2014, elucidated the genomic results of 3742 SNPs related to 300 genes in a large European-American population that had been exposed to traumatic events. They outlined an association between PTSD and SNPs pertaining to *SLC18A2* (VMAT2), a gene that codes for a protein to facilitate monoamine neurotransmitter carriage to synaptic vesicles [63]. In this study, SNP array delineated the common variation in twenty genes pertaining to PTSD. Mainly, the polymorphism in genes viz., *APOE, BDNF*, *COMT, FKBP5, HTR2A, SLC6A3, SLC6A4,* and TPH2 are associated with PTSD severity [63].

Glucocorticoids have a significant role in psychopathology of stress-related disorders including PTSD [64] (Figure 1). Glucocorticoids are metabolized by monoamine-metabolizing enzymes such as *cytochrome P450* (CYP), *11-β-hydroxysteroid dehydrogenase type 1* (*11βHSD1*), and 2 (*11βHSD2*) during PTSD-induced psychopathological cascades in CNS [64]. For instance, the damage to 5-HT neurons leads to ‘CYP3A overexpression’, which may be associated with high MAO-A activity and low plasma levels of 5-HT. Inhibition of cytochromes leads to the blockade of serotonin neuron excitations, which further exacerbates PTSD symptoms [64]. Furthermore, synthetic glucocorticoid like dexamethasone can enhance the upregulation of p11, known as S100A10-protein, which is normally reported to down-regulated in patients with depression, a common comorbid symptom in PTSD. This was confirmed by the studies on p11 Knockout mice as they exhibited depressive behavior in response to PTSD [65]. A few other research studies reported that rats exposed to imminent shock have elevated *p11* levels in prefrontal cortex. This study also reported that the synthetic glucocorticoid dexamethasone enhanced *p11* expression through glucocorticoid response elements (GREs) in the *p11* promoter, which has implications in psychopathology during PTSD [66]. Thus, the p11 protein plays a significant role in mood regulation during PTSD, and it is essential to develop novel therapeutic modalities to modulate the function of p11 in PTSD patients comorbid with major depressive disorder [65].

A substantial rise in catecholamines can induce the release of pro-inflammatory cytokines through the activation of nuclear factor- κB (NF-κB) and extracellular signal-regulated kinase (ERK), while stimulation of beta-2 adrenergic receptors leads to the secretion of IL-1β and IL-6 by macrophages and monocytes. The stimulation of the cholinergic anti-inflammatory agents inhibits the production of cytokines [47]. These pro-inflammatory factors confer a temporal relation between the inflammation and PTSD, suggesting the need to develop the novel pharmacological therapies to target inflammation by modulating catecholamine levels [45,67]. Dopamine is another monoaminergic catecholamine likely involved in the pathophysiology of PTSD. The 9R allele *SLC6A3* (solute carrier family 6 (neurotransmitter transporter, dopamine), member 3 locus is reported to be significant candidate gene to cause PTSD symptoms [68]. Hypermethylation of *SLC6A3* promoter locus could foster the risk of lifetime PTSD [26]. Increased dopamine transmission may compensate for the relative cortisol deficiency in PTSD [69]. On the other hand, GABAergic systems play a vital role in the pathophysiology of anxiety and depression, which are common symptoms in the people suffering from PTSD. Three polymorphisms in the a2 subunit of the GABA receptor (*GABRA2*) were found to have a significant association with childhood trauma [70].

Neuropeptide Y gene expression has implications in PTSD-induced psycho-pathophysiology [71]. In the hypothalamus, this neuropeptide Y is involved in impairing Corticotropin-releasing hormone (CRH) secretion by both paraventricular nucleus and in the noradrenergic neurons of the locus coeruleus, consequently mitigating EPSPs (excitatory postsynaptic potential) through the activation of presynaptic Y2 receptors [71]. The development of PTSD and comorbid depression typically is associated with stress-induced deficiency of neuropeptide Y (NPY). Hence, the strategies to enhance the production of NPY or the intranasal delivery of NPY is a promising approach to lessen neuroendocrine and molecular-behavioral impairments in PTSD models [71].

## 5. Metabolic Changes and PTSD

Patients with PTSD often have elevated BMI, glucose, insulin, and creatinine, which may be associated with cardiometabolic dysfunction [30] (Figure 1). For example, macrophages accumulate inside adipose tissue during obesity/insulin resistance generating cytokines and adipokines that may lead to inflammation [72]. Hyperinsulinemia sensitizes adipose tissue to lipid synthesis, which is associated with increased activity of glucocorticoids [66].

Fatty acids such as *linolenic, linoleate, docosahexaenoic, eicosapentaenoic*, and docosapentaenoic play crucial roles in neuroprotection. These were found to be reduced in the plasma of PTSD patients [73]. Normally, these fatty acids can confer changes inside the brain physiology through their functional implications in distinct ways. For example, they can affect the ‘structure of neuronal membranes, inflammation process, and HPA axis signaling’ and mitigate the oxidative stress [74]. They typically bind to the PPARγ or RXR in the nuclei of microglia to mitigate the expression of IL-6, IL-1β, iNOS, TNF-α, COX-2, and to alleviate reactive oxygen species (ROS) generation blocking NF-kB activity [74]. Inflammatory mediators derived from *omega-3* and *omega-6* fatty acid metabolism have shown opposite effects such as “anti-inflammatory, pro-inflammatory” effects. Lower concentrations of *omega-3* fatty acids and relatively higher concentrations of *omega-6* fatty acids lead to hyper-activation of the HPA axis and induce extensive rise in the cortisol levels. This rise in cortisol levels further reduces omega-3 concentrations through oxidative stress, which consequently enhances in the psychopathology of PTSD [74,75]. Apolipoprotein E (*ApoE*) regulates binding of lipoproteins to the low-density lipoprotein receptor and also regulates neuronal or glial responses to stress. *ApoE* gene is polymorphic and is reported to be involved in lessening memory impairment and relieving the individuals from traumatic memories during PTSD [70,76]. On the contrary, *ApoE2* also is involved in fostering behavioral, cognitive, and neuroendocrine changes associated with PTSD [77]. Hence, the dietary modalities rich in omega-3/omega-6 fatty acids may benefit PTSD patients by reducing traumatic memories and enhancing the cognitive or behavioral aspects through the modulation of metabolic cascades. However, further research is required to ascertain the complete role of fatty acid mediated metabolic alterations in PTSD patients.

## 6. Neuroendocrine Disturbances and PTSD

Neuroendocrine disturbances in the hypothalamus-pituitary-adrenal axis (HPA) could contribute to the psychopathology of PTSD [78] (Figure 1). The metabolic phenotype of PTSD may be caused partially by glucocorticoid hypersensitivity, which may affect inflammation, insulin resistance, oxidative stress, and energy deficiency [79]. PTSD patients have suppressed HPA axis activity, which progressively leads to increased catecholamine levels, corticotropin-releasing factor (CRF), and reduced cortisol levels [45,47,80]. A report by Heim C et al., 2008, found that the childhood exposure to severe abuse leads to sustained stress responses through persistent CRF-receptor activity in the later life [81]. Additionally, CRF-1-mediated signaling may promote extensive release of corticotropins, which are associated with other symptoms such as “suicide” and “psychosis” during stress. CRF1 signaling is also reported to be involved in contributing to the depression-related cognitive dysfunction during PTSD [82]. Pituitary adenylate cyclase-activating polypeptide (PACAP) could foster the CRF secretion, which is further associated with stress incidence in PTSD conditions. The deletion of the PACAP receptor *PAC-R1* in mice concluded that this deletion could contribute to lessen anxiety responses and fear conditioning [43]. The novel antagonists to target CRF-1 receptors may deliver effective clinical outcomes for treating comorbid symptoms during PTSD [83,84,85,86].

Glucocorticoid receptor (GR) activity is involved mediating negative feedback mechanism of HPA axis to release corticosteroids during stressful events [78]. Higher GR expression and lower overall methylation levels of GR promoter regions are reported in PTSD conditions. According to these findings, methylation in the GR promoter in military veterans with PTSD are lower than in patients without PTSD, indicating pharmacological strategies to modulate the methylation at GR promoter may be effective [78,87]. Numerous polymorphisms of GR-receptor gene have been reported in patients with PTSD [88,89]. For instance, SNPs in Bcl-1 are linked to glucocorticoid levels released via HPA axis [90]. The carriers homozygous to Bcl-1 SNP (G allele) significantly contributed to the incidence of traumatic memories and stress levels rather than heterozygous carriers of PTSD individuals [87,90,91].

Steroid receptor encoding *FKBP5* gene (*FK506-binding protein 5*) is reported to be involved in the pathogenesis of stress-related disorders by altering GR sensitivity [92]. For instance, the common variants of this gene foster significantly higher FKBP5 protein expression, enhance GR resistance, and impair negative feedback mechanisms in HPA axis. This leads to slower retrieval of stress-induced cortisol levels, which further enhances the risk of acquiring PTSD symptoms [92,93,94]. Four SNPs in *FKBP5* have been shown to correlate with childhood abuse; these SNP variants are appropriate for predicting the development of PTSD and its comorbid symptoms among African-American adults. Polymorphisms in *FKBP5* (rs1360780) can increase the risk of stress-related mental disorders in adulthood due to the methylation of the *FKBP5 gene locus* occurring as a consequence of childhood-dependent traumatic stress [95].

PTSD comorbid with depression is characterized by hyperactivity of the HPA axis, partly caused by abnormal inhibition of feedback mechanisms by endogenous glucocorticoids [46]. Licznerski et. al., 2015, reported a decline in glucocorticoid kinase 1 (*Sgk1*) transcript levels in PTSD patients compared to a control group. *Sgk1* regulates numerous enzymes and transcription factors involved in inflammation, glucocorticoid signaling, and cell proliferation. Novel therapeutic strategies to suppress *Sgk1* may improve contextual memory signals during PTSD [96]. Normally, glucocorticoids are metabolized through the *cytochrome P450* and *11bHSD1* and *11bHSD2* [64]. The glucocorticoid regulation of *p11* is particularly interesting and this regulation plays a crucial role in psychopathology and neurophysiology of PTSD [65].

## 7. Mitochondria and PTSD

Mitochondria play a role in the psychopathology and pathophysiology of PTSD [97] (Figure 1). The mitochondria role is pertaining to the PTSD-mediated symptomatology such as avoidance, inflammation, fear, anxiety, synaptic plasticity, and steroidogenesis [97]. For instance, glucocorticoids typically affect three mitochondrial functions viz., oxidation levels, mitochondrial membrane potential, and calcium retention [98]. Subjects with PTSD are characterized by elevated levels of lactate and pyruvate and diminished levels of citrate. Increased lactate concentrations may have a synergistic anti-lipolytic effect, reducing the availability of long-chain fatty acids, which can lead to obesity. Insulin resistance can cause damage to the mitochondria. It is still to be ascertained whether these changes are due to the effects of PTSD or other risk factors [99].

Dysregulated energy metabolism in mitochondria progressively leads to bioenergetic impairment during PTSD. In addition, chronic low-grade inflammation can cause the development of PTSD. However, higher energy metabolism during PTSD may contribute to the changes in neuronal signals, which exaggerate fear expression [100]. Genomic analysis of SNPs throughout the mitochondrial genome has been investigated [101]. This research has documented the key role of mitochondria in influencing cellular stress responses to a number of external cues during PTSD [102]. Mitochondrial dysfunction has been associated with abnormalities in synaptic plasticity implicated in mood and other mental disorders [103]. Flaquer et al. reported a link between PTSD and two mitochondrial SNP (mtSNP) viz., mt8414C located in MT-ATP8 and mt12501G located in MT-ND5 [101]. Both genes are involved in the regulation of reactive oxygen species (ROS). ROS trigger the formation of the inflammasome (NLRP3), which could induce higher cytokine levels (e.g., IL1-β and IL-18) to promote neuroinflammation during PTSD [99].

Heteroplasmy of above two mitochondrial gene variants towards the minor alleles likely increases the risk of PTSD, which leads to new insights for innovative treatment options [101]. Additionally, the number of mitochondrial DNA (*mtDNAcn*) copies associated with mitochondrial biogenesis is reported to be lower in the combat-related PTSD subjects [104]. Uncontrolled stress reduces mitochondrial anti-apoptotic Bcl-2 levels in cortical neurons and promotes the excessive NF-kB signaling, which further impairs hippocampal neurogenesis implicated in PTSD [103,104].

## 8. Neurogenesis and PTSD

Corticosterone can induce lipid and protein oxidation and inhibits antioxidant enzymes in the rat hippocampus. These effects are associated with pyramidal cell damage and neuronal death, which in turn trigger memory impairment [47]. Pro-inflammatory cytokine levels generated during PTSD could promote the ‘impairment of neurogenesis’ through blockade of brain-derived neurotrophic factor (BDNF) signaling, which eventually facilitates the generation of inflammatory cytokines and neuronal apoptosis in stress conditions [47]. BDNF, a key regulator of neuronal plasticity, is known to be involved in hippocampal neurogenesis. Impairment of BDNF signaling could cause PTSD comorbid with depression and fear. SNPs of *Val66Met* genes panning the human BDNF are correlated with the psychopathology of PTSD. *Met* carriers have been shown to have significantly higher susceptibility to PTSD compared to *Val/Val* carriers [105].

## 9. Premature Aging and PTSD

Recent epidemiological studies have described the increased risk of premature aging in PTSD patients due to chronic exposure to stress [106,107]. G-protein signal regulator 2 (RGS2) is involved in learning and memory processes. The pathophysiological association of RGS2 (*rs4606*) and PTSD after a traumatic hurricane was found to be evident in high stress and low social support settings, suggesting that RGS2 (*rs4606*) gene may play a critical role in post-injury recovery [108]. Two epigenetically altered networks related to premature aging in PTSD patients have been reported. The primary network is telomere control in relation to signaling cascades such as *Wnt/β-catenin* and *p53*. Mitochondrial dysfunction is the second epigenetic network correlated to premature aging in PTSD veterans [106]. Childhood trauma patients with PTSD had abnormally short leukocyte telomeres, which perhaps accelerate biological aging [107]. The premature aging is another significant research area where the influence of PTSD psychopathology yet to be addressed.

## 10. PTSD Therapeutic Modalities—Recent Advances

The development of effective diagnostic and therapeutic interventions to treat PTSD is challenging because of the intricate psychopathology and diverse neurophysiological alterations reported in patients [109]. Over the past few decades, cognitive-behavioral therapies (CBT) with administration of selective serotonin reuptake inhibitors (SSRIs) have been shown to be effective for PTSD treatment [3]. Of course, there are other treatments that appear effective for some patients, such as hypnotherapy and psychodynamic therapy. However, these clinical reports are not supported by well-controlled studies [110].

The co-morbidities such as drug abuse and depression are often associated with PTSD further complicate the selection of therapies. Systematic efforts to choose combinations of psychological and drug-based therapies have been gaining importance to treat PTSD. PTSD-induced trauma, chronicity induced from the specific of PTSD, gender and patient’s exposure to the traumatic events, and age factors are important other factors in choosing a specific treatment modality [3]. It remains unclear why combat veterans with PTSD are more resistant to treatment modalities than the other PTSD types [110]. Combat-exposed veterans have been diagnosed with several other psychiatric disorders including anxiety, mood instability, depression, substance abuse, phobias, panic disorders, and other psychosomatic diseases that have been described as a complex PTSD [111,112]. Nevertheless, psychotherapeutic interventions and pharmacotherapy remain the preferred treatment options [3]. It may be conjectured that psychotherapeutic interventions are beneficial to address the initial stages of PTSD [109].

Pharmacotherapy is an effective approach preferred for treating combat-induced PTSD with varying comorbidities. In some cases, both therapeutic modalities are chosen to deal with chronic PTSD [113]. Sertaline and fluoxetine are selective serotonin reuptake inhibitors (SSRIs) that already are approved by the US Food and Drug Administration as effective medications for chronic PTSD [114]. SSRI therapy is preferred immediately after a traumatic injury occurs to alleviate the PTSD-induced symptoms [100]. The clinical efficacy of these antidepressants including fluoxetine is associated with their immunomodulatory properties. A decrease in TNF levels is associated with a good response to these antidepressants during PTSD treatment, while an increase in IL-6 exerts an unsatisfactory response [115]. Chronic fluoxetine treatment helps to prevent the dysregulation of energy metabolism and improves PTSD symptoms in shocked mice [100]. Treatment of rats with paroxetine is reported to improve energy metabolism in the PFC, hippocampus, striatum, and cortex. The modulation of energy metabolism is correlated with alleviation of PTSD symptomatology as noted above. Fluoxetine can protect neurons from perturbations of cellular metabolism through the improvement of stress-induced mitochondrial dysfunctions [100]. For Korean War veterans, mitrazapine was reported to be effective in treating chronic PTSD [116]. Olanzapine and fluphenazine also were shown to treat combat-related PTSD to mitigate PTSD symptomatology [117].

Propranolol, a beta-blocker, is prescribed for the treatment of cardiac arrhythmias and hypertension. This drug is prescribed for PTSD patients because it can prevent retrieval of emotionally disturbing memories of PTSD-inducing traumatic events. Thus, it may diminish the psychopathological and neurophysiological alterations from the onset of trauma-induced PTSD [118]. Exposure to the combat-related stimuli can induce both enhanced autonomic reactivity, analgesia, and conditioned fear stimuli in war veterans. Opioid-induced analgesia is reversed by the intake of naloxone hydrochloride in animal models and mitigated conditioned fear stimuli [119,120]. Naloxone has been shown to mitigate these combat-related PTSD effects like analgesia and associated symptomology [121].

Epigenetic changes in the expression of HPA-axis signaling molecules can modify the synthesis of key feedback regulators such as *corticotrophin* and other glucocorticoid. The epigenetic changes in the genes coding for serotonergic and dopaminergic signaling networks may serve as targets for new therapeutic methods for PTSD [122]. Other therapies may include ACE inhibitors, oxytocin, cannabinoids, and glucocorticoids induce anti-inflammatory effects and they could be preferred as the additional therapies to mitigate symptomatology during PTSD. SSRIs also have anti-inflammatory effects in addition to anti-depression effects to treat PTSD symptomology [45]. Long-term treatment with dimethyltryptamine (DMT) in low doses has a beneficial effect on reinforced learning and mitigates conditioned fear stimuli by reducing the hyper-excitability of pyramidal neurons in the PFC that project into basolateral amygdala. This drug molecule can ameliorate the severity of the response to fear stimuli during PTSD [123,124].

Certain therapies have failed in treating patients with chronic PTSD have been published. The reasons for failure include comorbid symptomatology such as bipolar depression, emotional suffering, cognitive impairment, and failure to extinguish traumatic memories. Hence, research and clinical trials are focused on the development of therapeutic molecules against PTSD to modulate the neuronal receptor physiology and the downstream cascades to improve cognition, mitigate depression and traumatic memories [25].

Cotinine is an alkaloid metabolite of nicotine. It is being studied because of its positive allosteric modulators that can bind to the allosteric sites of *a7nAChRs* and stabilize the active receptor signaling in the brain cells and reduce symptomatology during PTSD [25]. Cotinine treatment in mice models of PTSD is reported to be effective in mitigating fear stimuli responses, anxiety, and depressive behaviors. This is attributed to cotinine-mediated positive modulation of nAChR [25]. Ginkgo extract is reported to reduce mitochondrial free radical generation implicated in PTSD symptomology [125]. *Ginko biloba* extracts can exhibit potential anxiolytic and mild antidepressant effects through the reversible inhibition of both monoamine oxidase A and B in the brain [125]. Administration of *neuropeptide Y* into the brain has exerted both the anxiolytic, antidepressant effects during PTSD [71]. These pharmacodynamic therapeutic strategies are considered effective in conjunction with psychotherapy.

## 11. Conclusions

Efforts to identify specific genetic factors that predispose individuals to PTSD have identified several genes. Special attention has been paid to *RD2*, *DRD4* (dopamine receptor 2,4), *SLC6A3* (DAT1 transporter), *SLC6A4* (serotonin transporter), *HTR2* (serotonin receptor 2A), *FKBP5* (FK506 binding protein 5), *BDNF* (brain-derived neurotrophic factor), *NPY* (neuropeptide Y), *GCCR* (glucocorticoid receptor), *DBH* (dopamine β-hydroxylase), *CNR1* (cannabinoid receptor 1), *GABRA2* (GABAA receptor), *COMT* (Catechol-O-methyltransferase), *Apo-E* (Apolipoprotein E), and *RGS2* (Regulator of G-protein signaling 2). These factors have been correlated with psychopathology and neurophysiology of PTSD [60]. However, GWAS studies of PTSD among combat veterans reported that there aresignificant SNPs involved in promoting PTSD symptomatology [126,127]. The development of CNS drugs has been impeded by low success rates in clinical trials due to intricate psychopathology and neurophysiology of PTSD [128].

## 12. Future Perspectives

Currently, the number of people diagnosed with PTSD just in the United States is 24 million (~8% of Americans) and costs $43 billion per year. There are limited strategies for clinical diagnosis and treatment of PTSD, but increased preclinical and clinical research is necessary to develop an explanation for psychopathological and neurophysiological aspects, which would contribute to future development of novel pharmacological therapies. There are limited favorable clinical outcomes observed from the use of antidepressants, antipsychotics, monoamine oxidase inhibitors, beta-blockers, and benzodiazepines [82,129,130]. These kinds of pharmacological and therapeutic interventions with limited effectiveness elicit a low adherence to treatment in a wide array of patients across the globe [25,131,132].

Novel therapeutic agents must be developed to reduce neuro-inflammation and improve neurogenesis and mitochondrial physiology in neurons to benefit the PTSD patients. The inability to distinguish PTSD from other stressor-related disorders may be due to the limited laboratory and clinical biomarkers for distinguishing PTSD and the varying etiopathogenesis that lead to PTSD [130,131,132]. Future research should focus on pharmacogenomics and especially mitochondrial genomics to identify specific biomarkers of PTSD. Such research likely will reveal avenues to use genomic knowledge as a strategy to refine medications that are used as treatments, to improve their effectiveness, and to provide prophylactics [82,129,130]. Newly developed diagnostic protocols should allow clinicians to develop *“Pharmacogenetic Profiles”* to choose suitable effective therapies against PTSD [82]. Further, these strategies may avoid undesirable side effects or cumulative intoxication through selective medications at the right dosage at the right time for each patient [25,82,128,131].

## Abbreviations

PPARγ	peroxisome proliferator-activated receptors;
RXR	retinoid X receptor;
COX	cyclooxygenase;
nAChR	nicotinic acetylcholinergic receoptor;
ACE	angiotensin converting enzyme
